# Associations between season of conception and maternal and perinatal health: a prospective birth cohort study

**DOI:** 10.7189/jogh.15.04243

**Published:** 2025-09-01

**Authors:** Qingxiu Li, Yecheng Miao, Jiayi Chen, Qian Zhang, Bin Sun, Zhengqin Wu, Junwei Liu, Huimin Shi, Haiyan Gao, Wei Li, Wenjuan Liu, Yibing Zhu, Haibo Li

**Affiliations:** 1 Fujian Maternity and Child Health Hospital, College of Clinical Medicine for Obstetrics & Gynecology and Pediatrics, Fujian Medical University, Fuzhou, China; 2 Department of Epidemiology and Health Statistics, School of Public Health, Fujian Medical University, Fuzhou, China; 3 School of Clinical Medicine, Ningxia Medical University, Yinchuan, China; 4 Fujian Obstetrics and Gynecology Hospital, Fuzhou, China; 5 Fujian Children's Hospital, Fuzhou, China

## Abstract

**Background:**

Global maternal health progress stagnated during the Sustainable Development Goal era, while the impact of meteorological conditions on maternal-infant outcomes remains contentious. We aimed to investigate the relationship between the season of conception and adverse perinatal outcomes.

**Methods:**

We conducted a prospective cohort study, collecting basic demographic characteristics of pregnant women and estimating the season of conception based on the date of the last menstrual period. We did a follow-up until delivery to monitor pregnancy health issues, such as gestational diabetes mellitus (GDM), gestational hypertension (GH), premature rupture of membranes (PROM), and postpartum haemorrhage (PPH), as well as neonatal health indicators such as birth weight and other relevant outcomes.

**Results:**

We included data from 26 341 pregnant women in our analysis. The average age of pregnant women was 30.3 years (standard deviation (SD) = 4.0), and 60.9% were primiparas. Compared to conception in spring (reference group), conception in summer was associated with a 15% reduction in GDM risk (odds ratio (OR) = 0.85; 95% confidence interval (CI) = 0.77–0.94) and a 10% lower PROM risk (OR = 0.90; 95% CI = 0.82–0.99), but a 46% increased PPH risk (OR = 1.46; 95% CI = 1.07–1.99). Conceptions in autumn and winter demonstrated even more pronounced protective effects, with the former showing a GDM risk reduction of 23% (OR = 0.77; 95% CI = 0.70–0.85) and 26% lower GH risk (OR = 0.74; 95% CI = 0.61–0.90), and the latter a 14% GDM risk reduction (OR = 0.86; 95% CI = 0.79–0.94) and 20% lower GH risk (OR = 0.80; 95% CI = 0.68–0.96).

**Conclusions:**

We found that conception in spring was associated with an increased risk of GDM, GH, and PROM, while conception in summer was linked to a higher risk of PPH. However, the preliminary nature of our findings suggests that further research is needed to confirm causality and assess the feasibility of any potential interventions.

Maternal and foetal health remains a key global concern, with progress in reducing maternal mortality stagnating during the Sustainable Development Goal era (2016–2020), as an annual reduction rate dropped to −0.04% [1]. This necessitates an unprecedented 11.6% annual decrease to meet the 2030 target of 70 deaths per 100 000 live births, highlighting the urgent need to strengthen maternal and foetal health initiatives worldwide. Gestational complications may compromise foetal development through placental insufficiency during pregnancy and epigenetically predispose offspring to lifelong cardiometabolic and neurodevelopmental disorders, thus increasing neonatal and maternal mortality and morbidity [2,3]. Furthermore, adverse pregnancy outcomes, such as preterm birth (PTB) and small for gestational age (SGA), are now recognised as long-term risk factors for premature mortality in women [4]. Therefore, there is an urgent need to reduce the risks of adverse maternal-infant health outcomes by controlling risk factors and implementing monitoring and interventions for high-risk pregnancies.

As an integral component of the human living environment, seasonal variations may exert influences on pregnancy health [5]. A large-scale study involving 194 028 participants in Southwest China demonstrated that pregnancies conceived during spring, autumn, and winter exhibited a significantly higher risk of PTB compared to those conceived in summer [6]. Similarly, a nationwide study in the Northern Hemisphere revealed an elevated risk of hypertensive disorders of pregnancy among women conceiving between February and June, with amplified associations observed in subgroups diagnosed with gestational hypertension (GH) and pre-eclampsia [7]. A Norway-based study in 2023 demonstrated that the incidence of gestational diabetes mellitus (GDM) was highest when the pregnancy started during the winter and lowest when the pregnancy started during the summer [8]. Moreover, a scholar from the USA highlighted that birth weights are lower for deliveries in winter and summer months [9]. Although a certain amount of international evidence has been accumulated regarding the environmental health effects during pregnancy [3,10,11], existing findings exhibit marked spatiotemporal heterogeneity [7]. Region-specific evidence focussing on China's unique climatic features and population characteristics remains insufficient. Furthermore, the impact of meteorological conditions on maternal and infant health outcomes remains contentious in current research [12,13]. For instance, an experimental study indicated that glucose and insulin are affected by environmental temperature, with elevated serum glucose and insulin in hot environments [14]. However, a study in temperate coastal regions of Australia found no clinically significant association between climate change, including temperature and GDM incidence [15]. The relationships between meteorology and pregnancy outcomes are not well known [9]. Current studies have predominantly focussed on specific pregnancy complications or health indicators, such as low birth weight (LBW), GDM, and GH, while neglecting other critical obstetric anomalies, including placenta previa and premature rupture of membranes (PROM) [8,16,17].

Therefore, we aim to systematically investigate the association between the season of conception and maternal-infant health outcomes and provide some insights for assessing the applicability of existing research findings within Chinese geographical contexts.

## METHODS

### Study population

**Figure 1:**
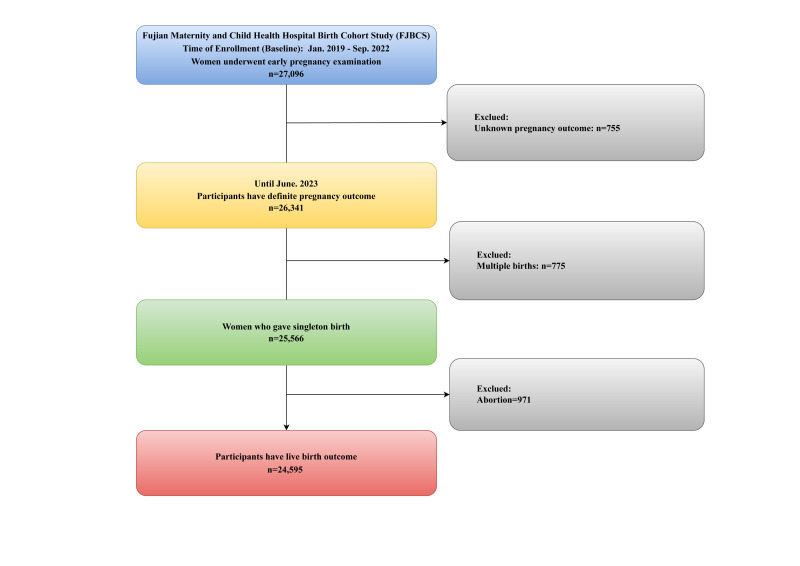
Flowchart of study participants.

We recruited a prospective birth cohort, the Fujian Maternity and Child Health Hospital Birth Cohort Study, at the Fujian Maternal and Child Health Hospital affiliated with Fujian Medical University, China. We enrolled 27 096 women undergoing early pregnancy examinations between January 2019 and September 2022. By June 2023, pregnancy outcomes were confirmed for 26 341 participants; 755 women with unknown outcomes were excluded. We also excluded women who had multiple births (n = 775), and those who had abortions (n = 971) were retained in the cohort for specific risk analyses but excluded from the live birth analyses (**Figure 1**). We obtained written informed consent from all participants prior to their inclusion. We conducted this study following the STROBE guidelines (Table S7 in the **Online Supplementary Document**) [18].

### Ascertainment of season of conception

We initially estimated the gestational age based on the last menstrual period and subsequently corrected it using foetal ultrasound measurements. The year is divided by astronomical seasons, which are based on the sunshine duration and solar altitude angle, into four seasons: spring (21 March to 20 June), summer (21 June to 20 September), autumn (21 September to 20 December), and winter (21 December to 20 March).

### Ascertainment of outcomes

Outcome measures were pregnancy complications and adverse pregnancy outcomes. We defined pregnancy complications as GDM (*i.e.* hyperglycaemia is first diagnosed during pregnancy) [19], GH (*i.e.* blood pressure ≥140 mm Hg systolic or 90 mm Hg diastolic on two separate occasions at least four hours apart after 20 weeks of pregnancy when previous blood pressure was normal) [20], pre-eclampsia (*i.e.* systolic blood pressure of ≥140 mm Hg or diastolic ≥90 mm Hg after 20 weeks of gestation in a woman with previously normal blood pressure, accompanied by proteinuria of ≥0.3 g in a 24-hour urine collection) [21], intrahepatic cholestasis of pregnancy (*i.e.* pregnancy-related pruritus with elevated serum aminotransferase or bile acids, after excluding other liver abnormalities) [22], caesarean delivery (*i.e.* a surgical procedure to deliver a foetus through laparotomy and hysterotomy), placenta previa (*i.e.* the complete or partial covering of the internal *os* of the cervix with the placenta), placental abruption (*i.e.* a large, clinically significant retroplacental hematoma), postpartum haemorrhage (PPH) (*i.e.* cumulative blood loss of >1000 mL or blood loss accompanied by signs and symptoms of hypovolemia), and abortion (*i.e.* the termination of pregnancy before the foetus reaches viability). We defined adverse pregnancy outcomes as SGA (*i.e.* a neonate whose birth weight is <10th percentile for gestational age, based on population-specific or customised growth standards), large for gestational age (LGA) (*i.e.* a neonate whose birth weight is >90th percentile for gestational age, based on standardised growth curves), LBW (*i.e.* a neonate with a birth weight less <2500 g), macrosomia (*i.e.* a neonate with a birth weight ≥4000 g), PTB (*i.e.* delivery before 37 completed weeks), PROM (*i.e.* rupture of membranes before labour), foetal distress (*i.e.* a syndrome where acute or chronic hypoxia in the womb endangers the health and life of the foetus) [23], and birth defects (*i.e.* a diverse range of conditions present from birth as well as minor conditions that do not pose a significant health issue) [24]. Regarding the diagnosis of SGA and LGA, we referred to the reference standard for the weight of infants of different gestational ages in China published in 2015 [25]. We based the definitions of caesarean delivery, placenta previa, placental abruption, PPH, abortion, SGA, LGA, LBW, macrosomia, PTB, and PROM on Williams Obstetrics [26]. In addition, we also followed up with the participants to obtain information on neonatal length and five-minute Apgar score.

### Ascertainment of covariates

All participants completed a questionnaire, and a trained staff member conducted an in-person interview with them. The questionnaire included questions on maternal and paternal demographics, including maternal age, paternal age, marital status, maternal smoking, assisted reproduction, pre-pregnancy body mass index (BMI), gravidity, parity, maternal educational level, and maternal alcohol consumption. Professional obstetricians collected self-reported data on marital status, maternal smoking, pre-pregnancy BMI, gravidity, parity, maternal educational level, and maternal alcohol consumption during early pregnancy. We computed the pre-pregnancy BMI using the self-reported pre-pregnancy weight divided by height squared (kg/m^2^). We classified educational levels as primary, secondary, and university (college and above) according to the degree of academic completion. We considered participants to be alcohol consumers if they drank alcohol >3 times per week before pregnancy and smokers if they consumed tobacco before or during pregnancy. Additionally, we obtained information on foetal sex and gestational age at delivery after delivery.

### Statistical analysis

We used means (standard deviations (SDs)) to describe continuous and percentages to describe categorical variables. We evaluated the differences between groups using Student's *t* test, one-way analysis of variance, Kruskal-Wallis test, or Mann-Whitney U test for continuous variables, and the χ^2^ test for categorical variables. We used logistic regression models to estimate the odds ratio (OR) and 95% confidence interval (CI) of season of conception with maternal and foetal health outcomes. We adjusted the analyses for potential confounding factors, such as maternal age, paternal age, marital status, maternal smoking, assisted reproduction, pre-pregnancy BMI, gravidity, parity, maternal educational level, maternal alcohol consumption, foetal sex, or gestational age at birth. We also performed subgroup analyses on the effects of season of conception on GDM and GH, considering factors such as maternal age, paternal age, parity, pre-pregnancy BMI, and modes of conception. In addition, we applied the Benjamini-Hochberg method to control the false-positive rate in multiple testing. For the analytical strategy excluding women with multiple pregnancies, we conducted sensitivity analyses by including these subpopulations to validate the robustness of the findings. We performed an additional sensitivity analysis to assess the robustness of the findings to unmeasured confounding using the E-value methodology by VanderWeele and Ding [27]. We performed statistical analyses using *R*, version 4.2.2 (R Core Team, Vienna, Austria), with all tests being two-sided and a significance level of 0.05.

## RESULTS

### Descriptive characteristics

We included 26 341 pregnant women, with 97.1% having singleton pregnancies (**Table 1**). The mean maternal age was 30.3 years (SD = 4.0), and the mean paternal age was 31.9 years (SD = 4.6). Most participants were of Han ethnicity, both for pregnant women (97.7%) and their partners (98.2%). During various seasons of conception, 68.9–70.3% of pregnant women had a pre-pregnancy BMI of 18.5–24.0 kg/m^2^. Moreover, there were significant differences in maternal alcohol consumption, paternal alcohol consumption, paternal smoking, assisted reproduction, and number of foetuses among the different seasons of conception (all *P* < 0.05).

**Table 1:** Clinical and biochemical characteristics of the study patients*

	Total (n = 26 341)	Spring (n = 8181)	Summer (n = 5729)	Autumn (n = 4540)	Winter (n = 7891)	*P*-value
**Maternal age (years), x̄ (SD)**	30.3 (4.0)	30.3 (4.1)	30.5 (4.1)	30.5 (4.0)	30.1 (4.0)	<0.001
**Paternal age (years), x̄ (SD)**	31.9 (4.6)	31.9 (4.6)	32.1 (4.6)	32.0 (4.6)	31.7 (4.5)	<0.001
**Maternal race-Han**	25 747 (97.7)	7976 (97.5)	5596 (97.7)	4459 (98.2)	7716 (97.8)	0.097
**Paternal race-Han**	25 854 (98.2)	8028 (98.1)	5627 (98.2)	4455 (98.1)	7744 (98.1)	0.593
**Maternal educational level-university**	20 432 (77.6)	6333 (77.4)	4448 (77.6)	3572 (78.7)	6079 (77.0)	0.148
**Maternal smoking**	563 (2.1)	192 (2.3)	109 (1.9)	87 (1.9)	175 (2.2)	0.114
**Maternal alcohol consumption**	3529 (13.4)	1167 (14.3)	647 (11.2)	430 (9.5)	1285 (16.3)	<0.001
**Paternal alcohol consumption**	12 484 (47.4)	4047 (49.4)	2600 (45.4)	1904 (41.9)	3933 (49.8)	<0.001
**Paternal smoking**	8474 (32.2)	2724 (33.3)	1802 (31.5)	1387 (30.6)	2561 (32.5)	0.022
**Assisted reproduction**	2204 (8.4)	638 (7.8)	611 (10.7)	471 (10.4)	484 (6.1)	<0.001
**Number of foetuses**						0.011
1	25 566 (97.1)	7948 (97.2)	5531 (96.5)	4389 (96.7)	7698 (97.6)	
2	753 (2.9)	228 (2.8)	192 (3.4)	147 (3.2)	186 (2.4)	
≥3	22 (0.1)	5 (0.1)	6 (0.1)	4 (0.1)	7 (0.1)	
**Pre-pregnancy BMI (kg/m^2^)**						0.476
<18.5	3834 (15.2)	1218 (15.3)	871 (15.4)	669 (14.9)	1076 (15.1)	
18.5–24.0	17 589 (69.7)	5560 (70.0)	3926 (69.4)	3098 (68.9)	5005 (70.3)	
24.0–28.0	3080 (12.2)	952 (12.0)	696 (12.3)	585 (13.0)	847 (11.9)	
≥28.0	718 (2.8)	217 (2.7)	167 (3.0)	145 (3.2)	189 (2.7)	
**Gravidity**						0.444
1	11 663 (44.3)	3630 (44.4)	2474 (43.2)	2047 (45.1)	3512 (44.5)	
2	7775 (29.5)	2417 (29.5)	1703 (29.7)	1345 (29.6)	2310 (29.3)	
≥3	6903 (26.2)	2134 (26.1)	1552 (27.1)	1148 (25.3)	2069 (26.2)	
**Parity**						0.059
0	16 046 (60.9)	4976 (60.8)	3442 (60.1)	2841 (62.6)	4787 (60.7)	
1	9175 (34.8)	2860 (35.0)	2060 (36.0)	1517 (33.4)	2738 (34.7)	
≥2	1120 (4.3)	345 (4.2)	227 (4.0)	182 (4.0)	366 (4.6)	

BMI – body mass index

*Presented as n (%) unless specified otherwise.

### Relationship between the season of conception and pregnancy complications

**Figure 2:**
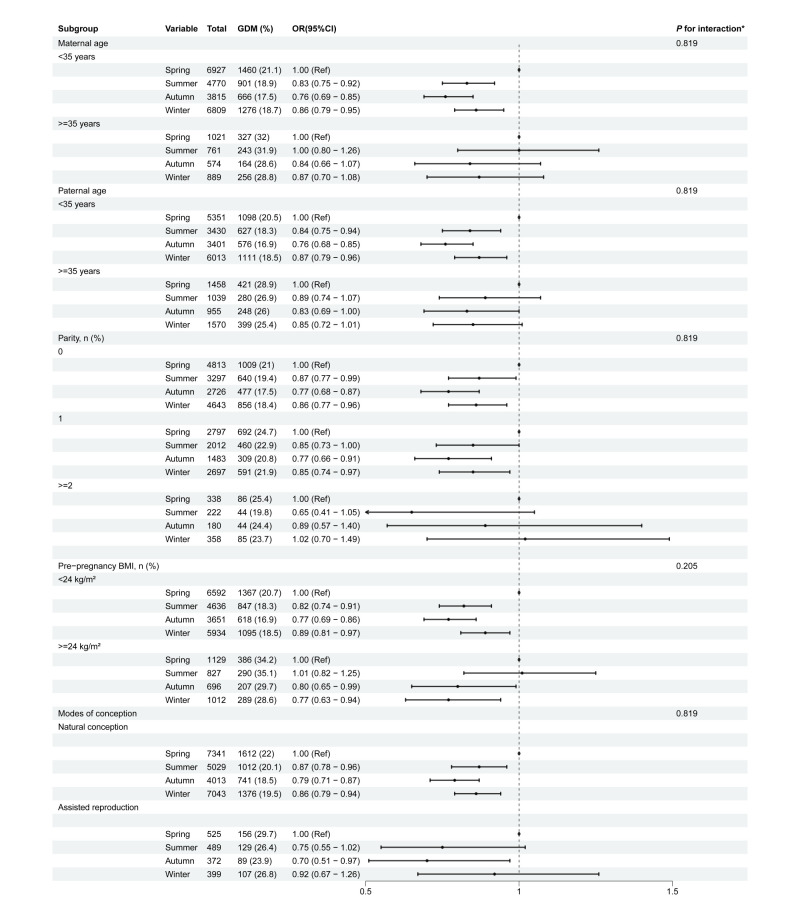
Subgroup analysis of the relative risk association between seasons of conception and GDM. Adjustment model: maternal age, paternal age, marital status, maternal smoking, assisted reproduction, pre-pregnancy BMI, gravidity, parity, maternal educational level, and maternal alcohol consumption. *P-values for interaction were adjusted using the Benjamini-Hochberg method. BMI – body mass index, CI – confidence interval, GDM – gestational diabetes mellitus, OR – odds ratio.

**Figure 3:**
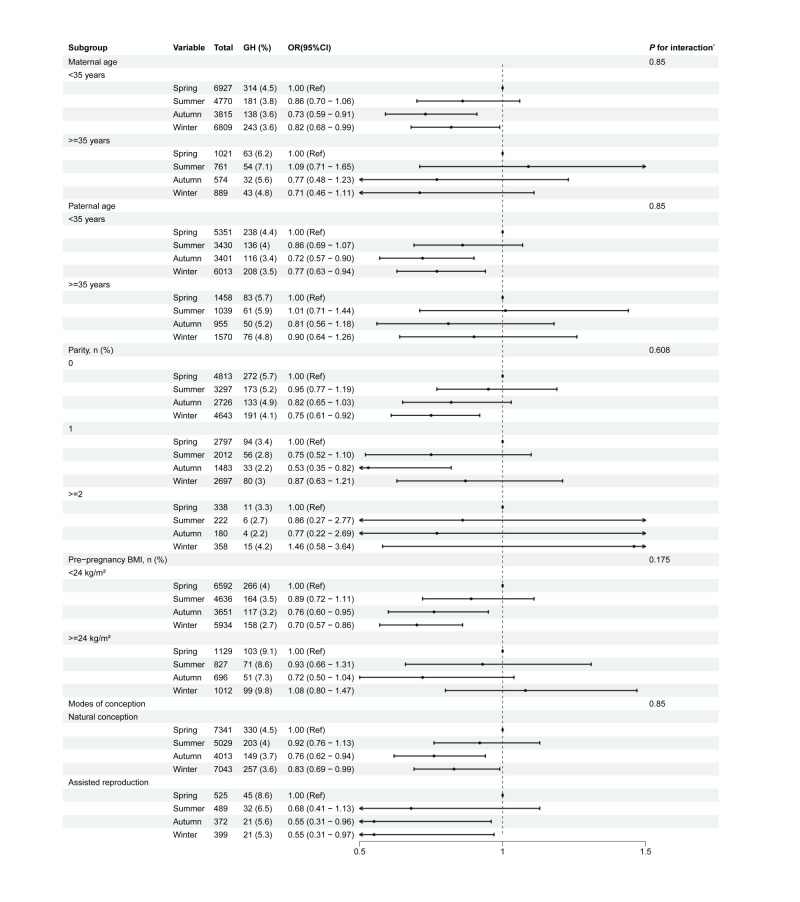
Subgroup analysis of the relative risk association between seasons of conception and GH. Adjustment model: maternal age, paternal age, marital status, maternal smoking, assisted reproduction, pre-pregnancy BMI, gravidity, parity, maternal educational level, and maternal alcohol consumption. *P-values for interaction were adjusted using the Benjamini-Hochberg method. BMI – body mass index, CI – confidence interval, GH – gestational hypertension, OR – odds ratio.

Pregnant women who conceived in the winter had the highest abortion rate (4.0%), and those who conceived in the spring and summer had the highest birth defect rate (6.7%) (Table S1 in the **Online Supplementary Document**). The incidence of modes of delivery (*P* = 0.009), GDM (*P* < 0.001), GH (*P* = 0.007), and PPH (*P* = 0.043) varied significantly across groups divided by season of conception (Table S2 in the **Online Supplementary Document**). Pregnant women who conceive in autumn and winter had a significantly lower risk of GDM and GH than those in spring (**Table 2**). Among these, conception in autumn had the greatest impact on the risk of GDM and GH, with the risk of GDM being 0.77 times that in spring (OR = 0.77; 95% CI = 0.70–0.85), and GH risk 0.74 times that of spring (OR = 0.74; 95%CI = 0.61–0.90). We additionally performed subgroup analyses, which revealed no interactions between conception season and maternal age, paternal age, parity, maternal pre-pregnancy BMI, or modes of conception in relation to GDM or GH risk (all *P* > 0.05) (**Figure 2**, **Figure 3**). Pregnant women conceiving in the summer were more likely to experience PPH than those who conceive in the spring (OR = 1.46; 95% CI = 1.07–1.99). However, no association was found between the season of conception and caesarean delivery, pre-eclampsia, intrahepatic cholestasis of pregnancy, placenta previa, and placental abruption.

**Table 2:** Association between season of conception and maternal pregnancy outcomes*

			Crude		Adjustment			
	**Total (n = 24 595)**	**Event (%)**	**OR (95% CI)**	***P-*value**	**OR (95% CI)**	***P-*value**	***P-*value†**	**E-value‡**
**GDM**								
Spring	7646	1784 (23.3)	ref		ref			
Summer	5322	1141 (21.4)	0.90 (0.82–0.98)	0.011	0.85 (0.77–0.94)	0.001	0.008	1.63
Autumn	4236	827 (19.5)	0.80 (0.73–0.87)	<0.001	0.77 (0.70–0.85)	<0.001	<0.001	1.92
Winter	7391	1527 (20.7)	0.86 (0.79–0.92)	<0.001	0.86 (0.79–0.94)	0.001	0.008	1.60
**GH**								
Spring	7646	374 (4.9)	ref		ref			
Summer	5322	231 (4.3)	0.88 (0.75–1.04)	0.144	0.89 (0.74–1.08)	0.233	0.923	
Autumn	4236	169 (4.0)	0.81 (0.67–0.97)	0.024	0.74 (0.61–0.90)	0.003	0.021	2.04
Winter	7391	282 (3.8)	0.77 (0.66–0.9)	0.001	0.80 (0.68–0.96)	0.013	0.091	1.81
**PE**								
Spring	7646	122 (1.6)	ref		ref			
Summer	5322	76 (1.4)	0.89 (0.67–1.19)	0.444	0.91 (0.66–1.25)	0.553	0.923	
Autumn	4236	73 (1.7)	1.08 (0.81–1.45)	0.600	1.03 (0.75–1.40)	0.869	0.869	
Winter	7391	87 (1.2)	0.73 (0.56–0.97)	0.029	0.82 (0.60–1.10)	0.187	0.949	
**ICP**								
Spring	7646	98 (1.3)	ref		ref			
Summer	5322	56 (1.1)	0.82 (0.59–1.14)	0.236	0.75 (0.52–1.09)	0.132	0.660	
Autumn	4236	55 (1.3)	1.01 (0.73–1.41)	0.938	0.95 (0.67–1.33)	0.754	0.869	
Winter	7391	101 (1.4)	1.07 (0.81–1.41)	0.649	0.99 (0.73–1.34)	0.949	0.949	
**Placenta previa**								
Spring	7646	46 (0.6)	ref		ref			
Summer	5322	34 (0.6)	1.06 (0.68–1.66)	0.790	0.98 (0.59–1.60)	0.923	0.923	
Autumn	4236	31 (0.7)	1.22 (0.77–1.92)	0.398	1.17 (0.73–1.89)	0.512	0.869	
Winter	7391	50 (0.7)	1.13 (0.75–1.68)	0.565	1.27 (0.83–1.95)	0.265	0.949	
**Placental abruption**								
Spring	7646	12 (0.2)	ref		ref			
Summer	5322	4 (0.1)	0.48 (0.15–1.48)	0.202	0.51 (0.14–1.90)	0.319	0.923	
Autumn	4236	3 (0.1)	0.45 (0.13–1.60)	0.217	0.52 (0.14–1.94)	0.334	0.869	
Winter	7391	10 (0.1)	0.86 (0.37–2.00)	0.729	0.96 (0.38–2.43)	0.934	0.949	
**PPH**								
Spring	7646	96 (1.3)	ref		ref			
Summer	5322	99 (1.9)	1.49 (1.12–1.98)	0.006	1.46 (1.07–1.99)	0.017	0.119	2.28
Autumn	4236	71 (1.7)	1.34 (0.98–1.83)	0.063	1.25 (0.90–1.72)	0.180	0.869	
Winter	7391	115 (1.6)	1.24 (0.95–1.63)	0.118	1.20 (0.89–1.61)	0.227	0.949	
**Caesarean delivery**								
Spring	7646	2768 (36.2)	ref		ref			
Summer	5322	2045 (38.4)	1.10 (1.02–1.18)	0.010	1.08 (0.99–1.17)	0.072	0.432	
Autumn	4236	1578 (37.3)	1.05 (0.97–1.13)	0.255	1.01 (0.93–1.10)	0.772	0.869	
Winter	7391	2635 (35.7)	0.98 (0.91–1.04)	0.482	1.00 (0.93–1.08)	0.910	0.949	

CI – confidence interval, GDM – gestational diabetes mellitus, GH – gestational hypertension, ICP – intrahepatic cholestasis of pregnancy, OR – odds ratio, PE – pre-eclampsia, PPH – postpartum haemorrhage, ref – reference

*Adjustment model: maternal age, paternal age, marital status, maternal smoking, assisted reproduction, pre-pregnancy BMI, gravidity, parity, maternal educational level, and maternal alcohol consumption.

†*P*-values were adjusted using the Benjamini-Hochberg method.

‡The E-value was not calculated due to a lack of statistical significance in the *P*-value.

### Association between different seasons of conception and foetal-neonatal outcomes

Excluding participants with miscarriage and non-singleton pregnancy, the average gestational age at delivery of the foetus was 39.2 years (SD = 1.5), the average birth weight was 3245.3 g (SD = 440.7), and there was a 7.2% incidence of SGA, 6.2% of LGA, 4.3% of LBW, and 3.6% of macrosomia (Table S3 in the **Online Supplementary Document**). Among the four seasons of conception, the probability of PTB in pregnancies conceived in autumn was the highest, followed by spring, and the lowest in summer and winter, while PROM and foetal distress were most common in women conceived in winter. After adjusting for confounding factors, including maternal age, paternal age, marital status, maternal smoking, assisted reproduction, pre-pregnancy BMI, gravidity, parity, maternal educational level, and maternal alcohol consumption, women who conceived in summer showed a decreased risk of PROM compared to those who conceived in spring (OR = 0.90; 95% CI = 0.82–0.99), while no significant association was detected between the season of conception and the risk of incidence of PTB and foetal distress (all *P* > 0.05) (**Table 3**). According to the results of regression analysis, additionally adjusted for foetal sex or gestational age at birth, no significant correlation was found between the season of conception and abnormal foetal birth weight.

**Table 3:** Association between season of conception and foetal-neonatal outcomes*

			Crude		Adjustment*			
	**Total (n = 24 595)**	**Event (%)**	**OR (95% CI)**	***P-*value**	**OR (95% CI)**	***P-*value**	***P-*value**†	**E-value‡**
**PROM**								
Spring	7646	1870 (24.5)	ref		ref			
Summer	5322	1214 (22.8)	0.91 (0.84–0.99)	0.030	0.90 (0.82–0.99)	0.031	0.217	1.46
Autumn	4236	1000 (23.6)	0.95 (0.87–1.04)	0.300	0.93 (0.85–1.02)	0.126	0.592	
Winter	7391	1752 (23.7)	0.96 (0.89–1.03)	0.281	0.94 (0.87–1.02)	0.16	0.744	
**Foetal distress**								
Spring	7646	535 (7.0)	ref		ref			
Summer	5322	352 (6.6)	0.94 (0.82–1.08)	0.395	0.94 (0.81–1.10)	0.464	0.950	
Autumn	4236	304 (7.2)	1.03 (0.89–1.19)	0.715	1.00 (0.85–1.16)	0.952	0.952	
Winter	7391	556 (7.5)	1.08 (0.96–1.22)	0.214	1.10 (0.96–1.25)	0.169	0.744	
**PTB**								
Spring	7646	451 (5.9)	ref		ref			
Summer	5322	308 (5.8)	0.98 (0.84–1.14)	0.791	1.02 (0.87–1.20)	0.789	0.950	
Autumn	4236	258 (6.1)	1.03 (0.88–1.21)	0.672	1.01 (0.86–1.19)	0.894	0.952	
Winter	7391	428 (5.8)	0.98 (0.86–1.12)	0.778	0.98 (0.84–1.13)	0.744	0.744	
**LBW**								
Spring	7646	303 (4.0)	ref		ref			
Summer	5322	195 (3.7)	0.87 (0.72–1.04)	0.127	0.88 (0.66–1.16)	0.371	0.950	
Autumn	4236	167 (3.9)	0.93 (0.76–1.12)	0.442	0.81 (0.61–1.08)	0.148	0.592	
Winter	7391	262 (3.5)	0.94 (0.79–1.11)	0.448	0.92 (0.72–1.19)	0.544	0.744	
**Macrosomia**								
Spring	7646	264 (3.5)	ref		ref			
Summer	5322	191 (3.6)	0.97 (0.81–1.18)	0.788	0.95 (0.77–1.18)	0.666	0.950	
Autumn	4236	128 (3.0)	0.82 (0.66–1.01)	0.063	0.82 (0.65–1.04)	0.100	0.592	
Winter	7391	205 (2.8)	0.84 (0.70–1.01)	0.068	0.88 (0.72–1.08)	0.226	0.744	
**SGA**								
Spring	7646	468 (6.1)	ref		ref			
Summer	5322	335 (6.3)	0.97 (0.84–1.12)	0.685	1.01 (0.85–1.19)	0.950	0.950	
Autumn	4236	294 (6.9)	1.06 (0.91–1.24)	0.435	1.06 (0.91–1.25)	0.450	0.952	
Winter	7391	458 (6.2)	1.07 (0.94–1.23)	0.304	1.13 (0.97–1.31)	0.109	0.744	
**LGA**								
Spring	7646	431 (5.6)	ref		ref			
Summer	5322	327 (6.1)	1.03 (0.89–1.19)	0.712	0.97 (0.82–1.15)	0.713	0.950	
Autumn	4236	217 (5.1)	0.85 (0.72–1.01)	0.061	0.84 (0.70–1.01)	0.058	0.406	
Winter	7391	379 (5.1)	0.96 (0.84–1.11)	0.614	0.94 (0.80–1.10)	0.438	0.744	

CI – confidence interval, LBW – low birth weight, LGA – large for gestational age, OR – odds ratio, PROM – premature rupture of membranes, PTB – preterm birth, ref – reference, SGA – small for gestational age

*Adjustment model: maternal age, paternal age, marital status, maternal smoking, assisted reproduction, pre-pregnancy BMI, gravidity, parity, maternal educational level, and maternal alcohol consumption (birth weight was additionally adjusted for foetal sex, and LBW and macrosomia were additionally adjusted for gestational age at delivery).

†*P*-values were adjusted using the Benjamini-Hochberg method.

‡The E-value was not calculated due to a lack of statistical significance in the *P*-value.

### Sensitivity analysis

To assess the robustness of the results, we conducted a sensitivity analysis (Tables S4–6 in the **Online Supplementary Document**). Results indicated that associations between conception season and risks of GDM, GH, PPH, and PROM remained.

## DISCUSSION

We observed a seasonal pattern in some maternal and perinatal health issues. The risk of GDM varied by conception season, with the highest risk observed in spring, followed by winter, summer, and autumn. Similarly, GH showed the highest incidence in spring, with decreasing risks in winter and autumn. Moreover, the likelihood of PROM was higher in pregnancies conceived in spring than in summer, while PPH was more common in pregnancies conceived in summer than in spring. These results imply that seasonal variations and the associated environmental variations may influence maternal and foetal health.

In contrast to a nationwide study in Norway on populations of European, Asian, and African descent [8], we found that, based on the season of conception, the incidence of GDM was the highest in spring and the lowest in autumn. Fujian is located on the southeastern edge of the Eurasian continent, facing the Pacific Ocean to the east, and has a typical subtropical monsoon climate characterised by prolonged sunlight exposure during summer and autumn [28]. Given that UV exposure represents the primary source of boosting serum vitamin D levels, the abundant sunlight in these seasons likely enhances vitamin D synthesis among pregnant women [29]. This elevated vitamin D status may consequently reduce plasma glucose levels at 60-minute during oral glucose tolerance tests, thereby contributing to a lower incidence of GDM [30]. In spring and winter, lower temperatures could lead to increased energy intake and reduced motivation for physical activity, resulting in weight gain during preconception or early pregnancy, possibly raising the risk of GDM [31,32]. However, with higher ambient temperatures in summer, blood flow redistribution between cutaneous and visceral vascular beds may elevate venous plasma glucose levels, which might explain the higher GDM risk in summer-conceiving women *vs.* those conceiving in autumn [32].

Consistent with an extensive cohort study from Australia, we found that GH rates were the lowest in autumn and the highest in spring by month of conception [5]. The seasonal variation in GH risk may be attributed to three factors. First, lower average temperatures in spring compared to autumn reduce the vasodilatory effects [33]. Second, during the rainy season (March to September), precipitation limits sunlight availability [34], which may impair maternal vitamin D synthesis [29]. Given the negative association between vitamin D and blood pressure levels [35], this may further increase the risk of GH. Third, insufficient sunlight is a recognised risk factor for hypertension [36]. It should be emphasised that the biological mechanisms we proposed, which include potential temperature-mediated effects on glucose metabolism and blood pressure regulation, along with the hypothesised association between vitamin D levels and seasonal patterns of GDM, are framed as hypotheses for future study rather than conclusions. A study involving 26 125 pregnant women from Recife, Brazil, reached a different conclusion from ours, indicating that the risk of hypertensive disorders of pregnancy, including GH, was higher in the cooler months [37]. A 2020 Denmark study reported that the risk of GH was the highest in pregnancies conceived during the summer and reached its nadir in late fall and winter [38]. The variations in study design, climatic conditions, socioeconomic level, lifestyles, and exposure periods may partially explain the inconsistency between the results of our study and those of others.

In addition to pregnancy complications, we also explored the association between conception season and other health issues during pregnancy, finding that PROM was more prevalent among pregnancies conceived in spring than in summer. At the same time, PPH incidence was higher in pregnancies conceived during summer compared to those conceived in spring. A 2024 study from Iran reached similar conclusions to ours, indicating that October had the highest number of referrals for obstetric haemorrhage [39]. Conversely, results from a study in Xinxiang, China suggested that high temperatures are associated with an increased risk of PROM, while cold temperatures may act as a protective factor against PROM [40]. Previous studies suggest that, in addition to temperature, particulate matter ≤2.5 μm, particulate matter ≤10 μm, sulphur dioxide, and carbon monoxide interact with each other to increase the risk of PROM [41]. Therefore, the difference in the results between the two places may also be affected by different degrees of environmental pollution caused by factors such as socioeconomic development and differences in topography and landforms [42]. It should be noted that the observed associations involving GH in winter-conceived pregnancies, PROM, and PPH may be subject to false positives attributable to multiple testing. Considering this, we strongly recommend that future research prioritise the use of prospective validation cohorts in conjunction with mechanistic studies. This approach will enable a more robust exploration of the association between the season of conception and adverse pregnancy outcomes.

Our study was a prospective cohort study with a large sample size, which enhances the reliability of the findings and allows for a more comprehensive assessment of the relationship between season of conception and maternal and infant health outcomes. In addition, we covered almost all possible adverse events for both pregnant women and foetuses throughout the pregnancy, compensating for the lack of research on the season of conception and maternal and infant health outcomes based on a subtropical monsoon climate. Nevertheless, there are still some limitations in the study. First, our single-centre observational study inherently presents limitations in cross-regional comparative analysis and comprehensive mechanistic investigation. Despite these constraints, the findings could offer insights for regions with comparable climatic conditions and provide directions for further mechanistic research. Second, due to limitations in data collection, we were unable to adjust for all confounding factors, such as social factors and individual behavioural variations. To address this concern, we adjusted for major confounding factors as much as possible and performed sensitivity analyses using the E-value methodology to assess the robustness of our findings to unmeasured confounding. These analyses indicated that our results are less likely to be reversed by unadjusted confounding factors. Third, certain data in the study were self-reported, and gestational age was inferred from the last menstrual period, potentially introducing bias. Notably, physicians recorded the self-reported data during initial prenatal visits in early pregnancy, significantly minimising recall and measurement bias. We also further employed foetal ultrasound to correct gestational age by re-estimating the last menstrual period, which allowed us to make more precise conception timing inferences. Furthermore, our data originated from medical diagnostic records, ensuring a certain level of reliability. Fourth, we employed astronomical seasons, which are less precise than directly using meteorological or photoperiod criteria. However, astronomical seasons reflect actual changes in sunshine duration and solar altitude angle and are easier for the public to understand and apply than meteorological or photoperiod criteria. Finally, we did not incorporate individual-level exposure assessments because the assessments present substantial methodological complexities involving intricate measurement protocols, and potential delays may occur in estimating health effects. Therefore, we used seasonal average meteorological data as proxies for environmental exposure to reflect overall effects in our study to ensure methodological feasibility and facilitate a more comprehensive understanding of the effect of environmental parameters on health-related outcomes. Nonetheless, future studies advancing individual-level exposure assessment remain essential to delineate precise biological pathways linking environmental factors to pregnancy outcomes.

## CONCLUSIONS

The incidence of GDM, GH, PROM, and PPH varies significantly across the season of conception. These patterns suggest that environmental factors, including temperature fluctuations and daylight exposure, may influence maternal physiology and contribute to differential pregnancy outcomes. Understanding the underlying mechanisms behind these seasonal influences could improve risk prediction and prevention strategies for adverse pregnancy outcomes, warranting further investigation in future research.

## Additional Material


Online Supplementary Document
