# Supplementary material for: Associations between season of conception and maternal and perinatal health: a prospective birth cohort study
Source: J Glob Health. 2025 Sep 1;15:04243. doi: 10.7189/jogh.15.04243 (PMC12400889; doi:10.7189/jogh.15.04243)
Supplement: Online Supplementary Document [file jogh-15-04243-s001.pdf]

Supplement to: Li Q, Miao Y, Chen J, Zhang Q, Sun B, Wu Z, Liu J, Shi H, Gao H, Li W, Liu W, Zhu Y, Li H. Associations between season of conception and maternal and perinatal health: a prospective birth cohort study. J Glob Health. 2025;15:04243.

Supplementary materials

Associations between Season of Conception and Maternal and Perinatal Health: A Prospective Birth Cohort Study

Qingxiu Li[1,2*], Yecheng Miao[3*], Jiayi Chen[1,2], Qian Zhang[2], Bin Sun[1], Zhengqin Wu[1,4], Junwei Liu[1,2], Huimin Shi[1,2], Haiyan Gao[1,4], Wei Li[1,4], Wenjuan Liu[5], Yibing Zhu[1‡], Haibo Li[1,2‡]

1 Fujian Maternity and Child Health Hospital, College of Clinical Medicine for Obstetrics & Gynecology and Pediatrics, Fujian Medical University, Fuzhou, China

2 Department of Epidemiology and Health Statistics, School of Public Health, Fujian Medical University, Fuzhou, China

3 School of Clinical Medicine, Ningxia Medical University, Yinchuan, China.

4 Fujian Obstetrics and Gynecology Hospital, Fuzhou, China.

5 Fujian Children's Hospital, Fuzhou, China.

[*] Joint first authorship.

[‡] Joint senior authorship.

Contents

Table S1 Relative risks of abortion and birth defects in subjects with the season of conception.

Table S2 Seasonal distribution of pregnancy complications.

Table S3 Fetal-neonatal outcomes with different seasons of conception.

Table S4 Relative risks of abortion and birth defects in subjects with the season of conception (participants including multiple pregnancies).

Table S5 Relationship between season of conception and maternal pregnancy outcomes (participants including multiple pregnancies).

Table S6 Association between season of conception and fetal-neonatal outcomes (participants including multiple pregnancies).

Table S1 Relative risks of abortion and birth defects in subjects with the season of conception.

Variable	Total (n = 25566)	Event (%)	Crude		Adjustment			
			OR (95%CI)	*P* value	OR (95%CI)	*P* value	*P* value *	E-value †
**Abortion**								
Spring	7948	302 (3.8)	1 (Ref)		1 (Ref)			
Summer	5531	209 (3.8)	0.99 (0.83~1.19)	0.950	1.06 (0.86~1.3)	0.586	0.802	-
Autumn	4389	153 (3.5)	0.91 (0.75~1.11)	0.376	0.99 (0.80~1.22)	0.908	0.908	-
Winter	7698	307 (4.0)	1.05 (0.89~1.24)	0.543	1.15 (0.96~1.38)	0.135	0.270	-
**Birth defects**								
Spring	7948	536 (6.7)	1 (Ref)		1 (Ref)			
Summer	5531	373 (6.7)	1.00 (0.87~1.15)	1.00	0.98 (0.84~1.14)	0.802	0.802	-
Autumn	4389	290 (6.6)	0.98 (0.84~1.13)	0.772	0.98 (0.84~1.14)	0.797	0.908	-
Winter	7698	498 (6.5)	0.96 (0.84~1.09)	0.489	1.03 (0.90~1.18)	0.693	0.693	-

CI – confidence interval, OR – odds ratio.

Adjustment model: maternal age, paternal age, marital status, maternal smoking, assisted reproduction, pre-pregnancy BMI, gravidity, parity, maternal educational level, and maternal alcohol consumption.

* *P* values were adjusted using the Benjamini-Hochberg method.

† The E-value was not calculated due to lack of statistical significance in the *P*-value.

Table S2 Seasonal distribution of pregnancy complications.

Variable	Total (n = 24595)	Spring (n = 7646)	Summer (n = 5322)	Autumn (n = 4236)	Winter (n = 7391)	*P* value
Modes of delivery, n (%)						0.009
Cesarean delivery	9026 (36.7)	2768 (36.2)	2045 (38.4)	1578 (37.3)	2635 (35.7)	
Normal delivery	15569 (63.3)	4878 (63.8)	3277 (61.6)	2658 (62.7)	4756 (64.3)	
GDM, n (%)	5279 (21.5)	1784 (23.3)	1141 (21.4)	827 (19.5)	1527 (20.7)	< 0.001
GH, n (%)	698 (2.8)	252 (3.3)	155 (2.9)	96 (2.3)	195 (2.6)	0.007
PE, n (%)	358 (1.5)	122 (1.6)	76 (1.4)	73 (1.7)	87 (1.2)	0.066
ICP, n (%)	310 (1.3)	98 (1.3)	56 (1.1)	55 (1.3)	101 (1.4)	0.458
Placenta previa, n (%)	161 (0.7)	46 (0.6)	34 (0.6)	31 (0.7)	50 (0.7)	0.851
Placental abruption, n (%)	29 (0.1)	12 (0.2)	4 (0.1)	3 (0.1)	10 (0.1)	0.474
PPH, n (%)	381 (1.5)	96 (1.3)	99 (1.9)	71 (1.7)	115 (1.6)	0.043

GDM – gestational diabetes mellitus, GH – gestational hypertension, ICP – intrahepatic cholestasis of pregnancy, PE – pre-eclampsia, PPH – Postpartum hemorrhage.

Table S3 Fetal-neonatal outcomes with different seasons of conception.

Variable	Total (n = 24595)	Spring (n = 7646)	Summer (n = 5322)	Autumn (n = 4236)	Winter (n = 7391)	*P* value
PROM, n (%)	5836 (23.7)	1870 (24.5)	1214 (22.8)	1000 (23.6)	1752 (23.7)	0.191
Fetal distress, n (%)	1747 (7.1)	535 (7.0)	352 (6.6)	304 (7.2)	556 (7.5)	0.254
Gestational age at delivery	39.2 ± 1.5	39.2 ± 1.5	39.2 ± 1.5	39.1 ± 1.5	39.2 ± 1.5	0.333
PTB, n (%)	1445 (5.9)	451 (5.9)	308 (5.8)	258 (6.1)	428 (5.8)	0.912
Baby sex-boys, n (%)	11990 (53.5)	3695 (53.2)	2643 (53.8)	2092 (53.5)	3560 (53.7)	0.799
Birth weight, Mean ± SD	3245.3 ± 440.7	3253.2 ± 448.3	3251.4 ± 439.0	3234.7 ± 438.1	3238.5 ± 435.1	0.077
Neonatal length, Mean ± SD	49.4 ± 3.6	49.3 ± 2.0	49.4 ± 1.9	49.5 ± 7.6	49.4 ± 1.9	0.206
5-minute Apgar score, Mean ± SD	10.0 ± 0.2	10.0 ± 0.1	10.0 ± 0.2	10.0 ± 0.1	10.0 ± 0.2	0.144
Gestational age-related birth weight categories, n (%)						0.259
SGA	1555 (7.2)	468 (7.0)	335 (6.8)	294 (7.4)	458 (7.5)	
AGA	18832 (86.6)	5810 (86.6)	4286 (86.6)	3436 (87.1)	5300 (86.4)	
LGA	1354 (6.2)	431 (6.4)	327 (6.6)	217 (5.5)	379 (6.2)	
LBW, n (%)	927 (4.3)	303 (4.5)	195 (3.9)	167 (4.2)	262 (4.3)	0.510
Macrosomia, n (%)	788 (3.6)	264 (3.9)	191 (3.9)	128 (3.2)	205 (3.3)	0.127

AGA – appropriate for gestational age, LGA – large for gestational age, LBW – low birth weight, PROM – premature rupture of membranes, PTB – preterm birth, SGA – small for gestational age.

Table S4 Relative risks of abortion and birth defects in subjects with the season of conception (participants including multiple pregnancies).

Variable	Total (n = 26431)	Event (%)	Crude		Adjustment			
			OR (95%CI)	*P* value	OR (95%CI)	*P* value	*P* value *	E-value †
**Abortion**								
Spring	8181	320 (3.9)	1(Ref)		1(Ref)			
Summer	5729	219 (3.8)	0.98 (0.82~1.16)	0.789	1.04 (0.85~1.27)	0.695	0.801	-
Autumn	4540	159 (3.5)	0.89 (0.73~1.08)	0.246	0.94 (0.77~1.16)	0.585	0.585	-
Winter	7891	324 (4.1)	1.05 (0.90~1.23)	0.530	1.14 (0.95~1.36)	0.156	0.312	-
**Birth defects**								
Spring	8181	586 (7.2)	1(Ref)		1(Ref)			
Summer	5729	424 (7.4)	1.04 (0.91~1.18)	0.594	0.98 (0.85~1.14)	0.801	0.801	-
Autumn	4540	318 (7.0)	0.98 (0.85~1.12)	0.739	0.96 (0.82~1.11)	0.559	0.585	-
Winter	7891	537 (6.8)	0.95 (0.84~1.07)	0.374	1.03 (0.90~1.17)	0.701	0.701	-

CI – confidence interval, OR – odds ratio.

Adjustment model: maternal age, paternal age, marital status, maternal smoking, assisted reproduction, pre-pregnancy BMI, gravidity, parity, maternal educational level maternal alcohol consumption, and number of the fetus.

* *P* values were adjusted using the Benjamini-Hochberg method.

† The E-value was not calculated due to lack of statistical significance in the *P*-value.

Table S5 Relationship between season of conception and maternal pregnancy outcomes (participants including multiple pregnancies).

Variable	Total (25319)	Event (%)	Crude		Adjustment			
			OR (95%CI)	*P* value	OR (95%CI)	*P* value	*P* value *	E-value †
**GDM**								
Spring	7861	1841 (23.4)	1(Ref)		1(Ref)			
Summer	5510	1182 (21.5)	0.89 (0.82~0.97)	0.007	0.84 (0.77~0.93)	<0.001	<0.001	1.67
Autumn	4381	873 (19.9)	0.81 (0.74~0.89)	<0.001	0.78 (0.71~0.86)	<0.001	<0.001	1.88
Winter	7567	1565 (20.7)	0.85 (0.79~0.92)	<0.001	0.86 (0.79~0.93)	<0.001	<0.001	1.60
**GH**								
Spring	7861	405 (5.2)	1(Ref)		1(Ref)			
Summer	5510	243 (4.4)	0.85 (0.72~1.00)	0.049	0.85 (0.71~1.02)	0.081	0.435	-
Autumn	4381	188 (4.3)	0.83 (0.69~0.99)	0.034	0.75 (0.62~0.90)	0.002	0.014	2.00
Winter	7567	308 (4.1)	0.78 (0.67~0.91)	0.001	0.82 (0.69~0.96)	0.017	0.119	1.74
**PE**								
Spring	7861	141 (1.8)	1(Ref)		1(Ref)			
Summer	5510	84 (1.5)	0.85 (0.65~1.11)	0.234	0.83 (0.62~1.12)	0.233	0.638	-
Autumn	4381	85 (1.9)	1.08 (0.83~1.42)	0.564	0.97 (0.73~1.29)	0.838	0.919	-
Winter	7567	105 (1.4)	0.77 (0.60~0.99)	0.045	0.84 (0.64~1.11)	0.222	0.934	-
**ICP**								
Spring	7861	106 (1.3)	1(Ref)		1(Ref)			
Summer	5510	63 (1.1)	0.85 (0.62~1.16)	0.297	0.78 (0.55~1.1)	0.154	0.616	-
Autumn	4381	63 (1.4)	1.07 (0.78~1.46)	0.684	0.98 (0.71~1.36)	0.919	0.919	-
Winter	7567	105 (1.4)	1.03 (0.78~1.35)	0.834	0.96 (0.72~1.28)	0.778	0.934	-
**Placenta previa**								
Spring	7861	51 (0.6)	1(Ref)		1(Ref)			
Summer	5510	36 (0.7)	1.01 (0.66~1.55)	0.974	0.93 (0.58~1.50)	0.782	0.782	-
Autumn	4381	34 (0.8)	1.20 (0.77~1.85)	0.417	1.13 (0.71~1.78)	0.608	0.919	-
Winter	7567	53 (0.7)	1.08 (0.73~1.59)	0.695	1.24 (0.82~1.87)	0.299	0.934	-
**Placental abruption**								
Spring	7861	12 (0.2)	1(Ref)		1(Ref)			
Summer	5510	4 (0.1)	0.48 (0.15~1.47)	0.198	0.51 (0.14~1.90)	0.319	0.638	-
Autumn	4381	3 (0.1)	0.45 (0.13~1.59)	0.214	0.52 (0.14~1.94)	0.334	0.919	-
Winter	7567	10 (0.1)	0.87 (0.37~2.00)	0.736	0.96 (0.38~2.43)	0.934	0.934	-
**PPH**								
Spring	7861	100 (1.3)	1(Ref)		1(Ref)			
Summer	5510	101 (1.8)	1.45 (1.10~1.91)	0.009	1.40 (1.03~1.89)	0.032	0.224	2.15
Autumn	4381	75 (1.7)	1.35 (1.00~1.83)	0.050	1.25 (0.91~1.71)	0.169	0.919	-

Winter	7567	118 (1.6)	1.23 (0.94~1.61)	0.131	1.18 (0.89~1.58)	0.252	0.934	-
**Cesarean delivery**								
Spring	7861	2965 (37.7)	1(Ref)		1(Ref)			
Summer	5510	2210 (40.1)	1.11 (1.03~1.19)	0.005	1.07 (0.99~1.17)	0.087	0.435	-
Autumn	4381	1711 (39.1)	1.06 (0.98~1.14)	0.144	1.01 (0.93~1.10)	0.766	0.919	-
Winter	7567	2785 (36.8)	0.96 (0.90~1.03)	0.241	1.00 (0.93~1.07)	0.932	0.934	-

CI – confidence interval, GDM – gestational diabetes mellitus, GH – gestational hypertension, ICP – intrahepatic cholestasis of pregnancy, OR – odds ratio, PE – pre-eclampsia, PPH – postpartum hemorrhage.

Adjustment model: maternal age, paternal age, marital status, maternal smoking, assisted reproduction, pre-pregnancy BMI, gravidity, parity, maternal educational level, and maternal alcohol consumption.

For the purpose of avoiding potential confounding effects of miscarriage on the occurrence of perinatal complications, women who experienced miscarriage were excluded from this part of the analysis.

* *P* values were adjusted using the Benjamini-Hochberg method.

† The E-value was not calculated due to lack of statistical significance in the *P*-value.

Table S6 Association between season of conception and fetal-neonatal outcomes (participants including multiple pregnancies).

Variable	Total (25319)	Event (%)	Crude		Adjustment			
			OR (95%CI)	*P* value	OR (95%CI)	*P* value	*P* value *	E-value †
**PROM**								
Spring	7861	1906 (24.2)	1(Ref)		1(Ref)			
Summer	5510	1249 (22.7)	0.92 (0.84~0.99)	0.034	0.91 (0.83~0.99)	0.033	0.099	1.43
Autumn	4381	1028 (23.5)	0.96 (0.88~1.04)	0.332	0.93 (0.85~1.02)	0.144	0.432	-
Winter	7567	1791 (23.7)	0.97 (0.90~1.04)	0.401	0.95 (0.87~1.02)	0.174	0.356	-
**Fetal distress**								
Spring	7861	547 (7.0)	1(Ref)		1(Ref)			
Summer	5510	362 (6.6)	0.94 (0.82~1.08)	0.380	0.94 (0.81~1.10)	0.463	0.843	-
Autumn	4381	315 (7.2)	1.04 (0.90~1.20)	0.631	1.00 (0.86~1.16)	0.978	0.978	-
Winter	7567	570 (7.5)	1.09 (0.96~1.23)	0.169	1.09 (0.96~1.25)	0.178	0.356	-
**PTB**								
Spring	7861	549 (7.0)	1(Ref)		1(Ref)			
Summer	5510	393 (7.1)	1.02 (0.89~1.17)	0.741	1.02 (0.87~1.19)	0.843	0.843	-
Autumn	4381	334 (7.6)	1.10 (0.95~1.27)	0.190	1.05 (0.90~1.22)	0.574	0.978	-
Winter	7567	520 (6.9)	0.98 (0.87~1.11)	0.784	1.00 (0.87~1.15)	0.978	0.978	-

CI – confidence interval, OR – odds ratio, PROM – premature rupture of membranes, PTB – preterm birth.

Adjustment model: maternal age, paternal age, marital status, maternal smoking, assisted reproduction, pre-pregnancy BMI, gravidity, parity, maternal educational level, maternal alcohol consumption and number of the fetus.

For the purpose of avoiding potential confounding effects of miscarriage on the occurrence of perinatal complications, women who experienced miscarriage were excluded from this part of the analysis.

Due to the poor comparability of fetal weights between multiple and singleton pregnancies, fetal weight analysis was not performed in this section.

**P* values were adjusted using the Benjamini-Hochberg method.

† The E-value was not calculated due to lack of statistical significance in the *P*-value.

STROBE Statement—Checklist of items that should be included in reports of ***cohort studies***

	Item No	Recommendation
**Title and abstract**	1	(*a*) Indicate the study's design with a commonly used term in the title or the abstract
		(*b*) Provide in the abstract an informative and balanced summary of what was done and what was found
**Introduction**		
Background/rationale	2	Explain the scientific background and rationale for the investigation being reported
Objectives	3	State specific objectives, including any prespecified hypotheses
**Methods**		
Study design	4	Present key elements of study design early in the paper
Setting	5	Describe the setting, locations, and relevant dates, including periods of recruitment, exposure, follow-up, and data collection
Participants	6	(*a*) Give the eligibility criteria, and the sources and methods of selection of participants. Describe methods of follow-up
		(*b*) For matched studies, give matching criteria and number of exposed and unexposed
Variables	7	Clearly define all outcomes, exposures, predictors, potential confounders, and effect modifiers. Give diagnostic criteria, if applicable
Data sources/ measurement	8*	For each variable of interest, give sources of data and details of methods of assessment (measurement). Describe comparability of assessment methods if there is more than one group
Bias	9	Describe any efforts to address potential sources of bias
Study size	10	Explain how the study size was arrived at
Quantitative variables	11	Explain how quantitative variables were handled in the analyses. If applicable, describe which groupings were chosen and why
Statistical methods	12	(*a*) Describe all statistical methods, including those used to control for confounding
		(*b*) Describe any methods used to examine subgroups and interactions
		(*c*) Explain how missing data were addressed
		(*d*) If applicable, explain how loss to follow-up was addressed
		(*e*) Describe any sensitivity analyses
**Results**		
Participants	13*	(a) Report numbers of individuals at each stage of study—eg numbers potentially eligible, examined for eligibility, confirmed eligible, included in the study, completing follow-up, and analysed
		(b) Give reasons for non-participation at each stage
		(c) Consider use of a flow diagram
Descriptive data	14*	(a) Give characteristics of study participants (eg demographic, clinical, social) and information on exposures and potential confounders
		(b) Indicate number of participants with missing data for each variable of interest
		(c) Summarise follow-up time (eg, average and total amount)
Outcome data	15*	Report numbers of outcome events or summary measures over time
Main results	16	(*a*) Give unadjusted estimates and, if applicable, confounder-adjusted estimates and their precision (eg, 95% confidence interval). Make clear which confounders were adjusted for and why they were included
		(*b*) Report category boundaries when continuous variables were categorized
		(*c*) If relevant, consider translating estimates of relative risk into absolute risk for a meaningful time period

Other analyses	17	Report other analyses done—eg analyses of subgroups and interactions, and sensitivity analyses
**Discussion**		
Key results	18	Summarise key results with reference to study objectives
Limitations	19	Discuss limitations of the study, taking into account sources of potential bias or imprecision. Discuss both direction and magnitude of any potential bias
Interpretation	20	Give a cautious overall interpretation of results considering objectives, limitations, multiplicity of analyses, results from similar studies, and other relevant evidence
Generalisability	21	Discuss the generalisability (external validity) of the study results
**Other information**		
Funding	22	Give the source of funding and the role of the funders for the present study and, if applicable, for the original study on which the present article is based

*Give information separately for exposed and unexposed groups.

Note: An Explanation and Elaboration article discusses each checklist item and gives methodological background and published examples of transparent reporting. The STROBE checklist is best used in conjunction with this article (freely available on the Web sites of PLoS Medicine at http://www.plosmedicine.org/, Annals of Internal Medicine at http://www.annals.org/, and Epidemiology at http://www.epidem.com/). Information on the STROBE Initiative is available at http://www.strobe-statement.org.
